# AIP1 suppresses neovascularization by inhibiting the NOX4-induced NLRP3/NLRP6 imbalance in a murine corneal alkali burn model

**DOI:** 10.1186/s12964-022-00877-5

**Published:** 2022-05-06

**Authors:** Qingyu Li, Xia Hua, Liangpin Li, Xueyan Zhou, Ye Tian, Yang Deng, Min Zhang, Xiaoyong Yuan, Wei Chi

**Affiliations:** 1 Clinical College of Ophthalmology, Tianjin Medical University, Tianjin, China; 2 Tianjin Key Laboratory of Ophthalmology and Visual Science, Tianjin Eye Institute, Tianjin Eye Hospital, Tianjin, China; 3 Tianjin Aier Eye Hospital, Tianjin University, Tianjin, China; 4 School of Medicine, Nankai University, Tianjin, China; 5 State Key Laboratory of Ophthalmology, Zhongshan Ophthalmic Center, Sun Yat-Sen University, Guangdong Provincial Key Laboratory of Ophthalmology and Visual Science, Guangzhou, 510060 China

**Keywords:** AIP1, Alkali burn, Corneal neovascularization, NADPH oxidase, NLRP3, NLRP6, ROS

## Abstract

**Background:**

Apoptosis signal-regulating kinase 1-interacting protein 1 (AIP1) participates in inflammatory neovascularization induction. NADPH oxidase 4 (NOX4) produces reactive oxygen species (ROS), leading to an imbalance in nucleotide-binding oligomerization domain-like receptor family pyrin domain containing 3 (NLRP3) and NLR family pyrin domain containing 6 (NLRP6) expression. The mechanisms of AIP1, NOX4, ROS and inflammasomes in corneal neovascularization were studied herein.

**Methods:**

C57BL/6 and AIP1-knockout mice were used in this study. The alkali burn procedure was performed on the right eye. Adenovirus encoding AIP1 plus green fluorescence protein (GFP) (Ad-AIP1-GFP) or GFP alone was injected into the right anterior chamber, GLX351322 was applied as a NOX4 inhibitor, and then corneal neovascularization was scored. The expression of related genes was measured by quantitative real-time polymerase chain reaction, western blotting and immunofluorescence staining. 2′,7′-Dichlorofluorescin diacetate staining was used to determine the ROS levels.

**Results:**

The expression of AIP1 was decreased, while that of cleaved interleukin-1β (clv-IL-1β) and vascular endothelial growth factor A (VEGFa) was increased after alkali burn injury. NOX4 expression was increased, the imbalance in NLRP3/NLRP6 was exacerbated, and corneal neovascularization was increased significantly in AIP1-knockout mice compared with those in C57BL/6 mice after alkali burns. These effects were reversed by AIP1 overexpression. NLRP3/NLRP6 expression was imbalanced after alkali burns. GLX351322 reversed the imbalance in NLRP3/NLRP6 by reducing the ROS levels. This treatment also reduced the expression of clv-IL-1β and VEGFa, suppressing neovascularization.

**Conclusions:**

AIP1 and NOX4 can regulate corneal inflammation and neovascularization after alkali burn injury. Based on the pathogenesis of corneal neovascularization, these findings are expected to provide new therapeutic strategies for patients.

**Plain English summary:**

Corneal alkali burn injury is a common type of ocular injury that is difficult to treat in the clinic. The cornea is a clear and avascular tissue. Corneal neovascularization after alkali burn injury is a serious complication; it not only seriously affects the patient’s vision but also is the main reason for failed corneal transplantation. Corneal neovascularization affects approximately 1.4 million patients a year. We show for the first time that AIP1 and NOX4 can regulate corneal inflammation and neovascularization after alkali burns. The expression of AIP1 was decreased, while that of clv-IL-1β and VEGFa was increased after alkali burns. We tried to elucidate the specific molecular mechanisms by which AIP1 regulates corneal neovascularization. NOX4 activation was due to decreased AIP1 expression in murine corneas with alkali burns. NOX4 expression was increased, the imbalance in NLRP3/NLRP6 was exacerbated, and corneal neovascularization was increased significantly in AIP1-knockout mice compared with those in C57BL/6 mice after alkali burns. These effects were reversed by AIP1 overexpression. Additionally, NLRP3/NLRP6 expression was unbalanced, with NLRP3 activation and NLRP6 suppression in the corneal alkali burn murine model. Eye drops containing GLX351322, a NOX4 inhibitor, reversed the imbalance in NLRP3/NLRP6 by reducing ROS expression. This treatment also reduced the expression of clv-IL-1β and VEGFa, reducing neovascularization. Therefore, we provide new gene therapeutic strategies for patients. With the development of neovascularization therapy, we believe that in addition to corneal transplantation, new drug or gene therapies can achieve better results.

**Video Abstract**

**Supplementary Information:**

The online version contains supplementary material available at 10.1186/s12964-022-00877-5.

## Background

The cornea is a clear and avascular tissue. An imbalance in pro- and antiangiogenic factors in corneal tissue leads to corneal neovascularization [1–3]. Angiogenesis is closely coordinated by a series of angiogenic cytokines, including vascular endothelial growth factor, basic fibroblast growth factor, transforming growth factor β and interleukin (IL)-1β [4] and antiangiogenic cytokines. Among them, vascular endothelial growth factor (VEGF) is a representative proangiogenic factor [5–7].

Inflammation is a common immune response to various infections or injuries and can cause various complex diseases. Inflammasomes are protein complexes that play critical roles in the inflammatory process, and the nucleotide-binding oligomerization domain-like receptor family pyrin domain containing 3 (NLRP3) inflammasome is the most widely studied. The NLRP3 inflammasome plays a crucial role in natural immunity [8–10]. NLRP3 is a NOD-like receptor (NLR) that forms a multiprotein complex with the core proteins caspase-1 and apoptosis-associated speck-like protein containing a CARD (ASC) [11, 12]. Inflammasome activation induces the release of substantial amounts of mature IL-1β. IL-1β can cause the release of VEGFa [13]. NLRP3 plays a crucial role in ocular neovascularization. In a corneal alkali burn model, alkali burn injury induces early innate immune activation in the cornea and disrupts the balance of NLRP3 and NLRP6 expression (14). However, the contributions of nucleotide-binding oligomerization domain (NOD)-like receptors (NLRs) to late corneal neovascularization after alkali burns remain unknown. Reducing the release of IL-1β and VEGFa by maintaining the balance of NLRP3 and NLRP6 is a critical strategy to alleviate corneal neovascularization.

Accumulating evidence shows that overexpression of NLRP3 inflammasome core molecules is induced by excess reactive oxygen species (ROS) in multiple animal models [15–18]. NADPH oxidase (NOX) activation can generate ROS, and this process is a crucial source of ROS [19–21]. The expression of NOX is increased in corneal epithelial cells after alkali burn injury. Diphenyleneiodonium chloride or apocynin, which are inhibitors of NOX, effectively attenuate alkali burn-induced ROS generation and reduce corneal neovascularization due to alkali burns [22]. However, the regulation of NLRs by NOX remains unclear. Our results suggest that the application of GLX351322, a NOX4 inhibitor, can inhibit NLRP3 activation, attenuate the inflammatory response and reduce corneal neovascularization by scavenging excess ROS.

Apoptosis signal-regulating kinase 1-interacting protein 1 (AIP1) was recently identified as a signalling scaffold protein. AIP1 downregulates various human cancers [23, 24]. In AIP1-KO mice, VEGF-induced neovascularization in the ear, cornea and retina was significantly enhanced [25]. Endothelial AIP1 regulates vascular remodelling by inhibiting NADPH oxidase-2 (NOX2) [26]. We aimed to elucidate the specific molecular mechanisms by which AIP1 regulates corneal neovascularization.

How AIP1 and NOX4 are associated with NLRP3/NLRP6-regulated corneal neovascularization is incompletely understood. The present study suggests that the protective effect of eye drops containing GLX351322 on corneal neovascularization after alkali burn injury may be related to the reduced ROS levels, the reversal of the NLRP3/NLRP6 imbalance, and the reduction in IL-1β and VEGFa production. AIP1 can attenuate corneal neovascularization via the NOX4-NLRP3/NLRP6-IL-1β-VEGFa pathway, acting in the same manner as GLX351322.

## Methods

### Animals and alkali burn mouse model

In this study, female C57BL/6 and AIP1-knockout (KO) mice (6–8 weeks old) were used. The mice were purchased from Jinan Pengyue Experimental Animal Breeding Co., Ltd. (Jinan, China) and Shanghai Southern Model Biology Research Centre (Shanghai, China) and were raised in the Experimental Animal Centre of Zhongshan Ophthalmic Centre, Sun Yat-sen University under specific-pathogen-free conditions. All the procedures involving animals were conducted strictly in accordance with the Association for Research in Vision and Ophthalmology (ARVO) Statement for the Use of Animals in Ophthalmic and Vision Research. All the animal experiments were approved by the Animal Care and Ethics Committee of the Zhongshan Ophthalmic Centre (Approval number: 2018-082). The mice were anaesthetized with an injection of 1% pentobarbital sodium (40–50 mg/kg), and a drop of 0.5% proparacaine was applied to the right corneal surface. The right eye was subjected to an alkali burn with Whatman filter paper (2 mm in diameter) soaked in 1 N NaOH. After placing the filter paper on the eye for 40 s, the eye was rinsed with 20 ml of 0.9% saline solution. Mouse corneas were monitored and photographed under a slit-lamp microscope (Topcon, Tokyo, Japan).

### Construction of adenoviral vectors

Adenoviral vectors encoding AIP1 and green fluorescent protein (Ad-AIP1-GFP) or a negative control (Ad-GFP) were constructed and purified by TranSheepBio (Shanghai, China). The viral titre was 1.27 × 10^11^ plaque-forming units (PFU)/ml, as provided by the company. Adenovirus encoding AIP1 plus GFP or GFP alone (2.54 × 10^8^ PFU) was injected into the right anterior chamber of the mice 2 days before alkali burn injury.

### Corneal whole mount staining

Ten days after alkali burn injury, the eyeballs were removed from the euthanized mice for corneal whole-mount staining [27, 28]. The stained corneas were observed under a fluorescence microscope (Olympus, Tokyo, Japan) at 40/100 × magnification.

### Evaluation of corneal neovascularization

The surfaces of the eyes were observed and assessed daily under a slit-lamp microscope (Topcon), and images were captured by an attached camera. Corneal opacity was graded on a scale of 0 to 4, and neovessel size was graded on a scale of 0 to 3, according to an accepted standard [29]. Two observers scored the corneal opacity and neovessel size independently, and the final score was the average of the scores.

## Quantitative real-time polymerase chain reaction (RT–qPCR)

The mice were sacrificed 10 days after alkali burn injury. The eyes were then enucleated from the euthanized mice, and the corneas were excised and dissected from the surrounding tissues. Sets of five corneas were prepared for each group. RNA was extracted using RNeasy MicroKit columns (Qiagen, Valencia, USA) according to the manufacturer’s instructions. After measuring the RNA concentration using a Nanodrop 2000 system (Thermo, Boston, USA), 1 µg of RNA was used to synthesize cDNA using HiScript II Reverse Transcriptase (Vazyme, Nanjing, China). The expression levels of AIP1, NOX4, NLRP3, NLRP6 and VEGFa were measured using the SYBR Green system (Roche, Pleasanton, USA). The cycle threshold for each mRNA was normalized to that of glyceraldehyde-3-phosphate dehydrogenase (GAPDH) mRNA and averaged. Each experiment was repeated five times independently. The sequences of the primers are listed in Table 1.

**Table 1:** The primer sequences of the related genes

GAPDH	
Forward primer sequence (5′–3′)	AAGAAGGTGGTGAAGCAGG
Reverse primer sequence (5′–3′)	GAAGGTGGAAGAGTGGGAGT
AIP1	
Forward primer sequence (5′–3′)	CTGGCACTTGAATAGGGTCT
Reverse primer sequence (5′–3′)	GGCTAAGGAGTAAGGAGGAAC
NOX4	
Forward primer sequence (5′–3′)	TGCCTGCTCATTTGGCTGT
Reverse primer sequence (5′–3′)	CCGGCACATAGGTAAAAGGATG
NLRP3	
Forward primer sequence (5′–3′)	ATTACCCGCCCGAGAAAGG
Reverse primer sequence (5′–3′)	TCGCAGCAAAGATCCACACAG
NLRP6	
Forward primer sequence (5′–3′)	CTCGCTTGCTAGTGACTACAC
Reverse primer sequence (5′–3′)	AGTGCAAACAGCGTCTCGTT
VEGFa	
Forward primer sequence (5′–3′)	GAGGTCAAGGCTTTTGAAGGC
Reverse primer sequence (5′–3′)	CTGTCCTGGTATTGAGGGTGG

### Western blotting

The mice were sacrificed 10 days after alkali burn injury. Sets of four corneas were prepared for each group. The corneas were lysed by ultrasonication (Sonifier 150; Branson, USA) using RIPA lysis buffer (P0013K; Beyotime, Shanghai, China) containing protease inhibitors. After agarose gel electrophoresis, the AIP1, NOX4, NLRP3, NLRP6, clv-casp1, ASC, clv-IL1β and actin levels were measured using mouse anti-AIP1 (1:100; Cat# sc-365921; Santa Cruz, Santa Cruz, USA), rabbit anti-NOX4 (1:500; Cat# A11274; ABclonal, Wuhan, China), rabbit anti-NLRP3 (1:500; Cat# A5652; ABclonal), rabbit anti-NLRP6 (1:500; Cat# A15628; ABclonal), rabbit anti-casp1 (1:200; Cat# A0964; ABclonal), rabbit anti-ASC (1:200; Cat# A1170; ABclonal), rabbit anti-IL1β (1:200; Cat# YT5201; Immunoway, USA) and mouse anti-actin (1:1000; Cat# RM2001; Ray, Beijing, China) primary antibodies, respectively. The polyvinylidene fluoride membrane (Millipore Immobilon-PSQ, USA) was then incubated with secondary antibodies for 1 h at room temperature. After the membrane was washed, the protein bands were detected using ultrasensitive enhanced chemiluminescence substrate (Boster, California, USA). The greyscale value for each protein was normalized to that of β-actin and averaged. Each experiment was repeated three times independently.

### In situ corneal ROS assay

The eyeballs in the twelve groups were embedded in Tissue Tek OCT compound (Sakura Finetek, Torrance, USA). After rewarming the unfixed frozen cross sections (7 μm) at room temperature for 1 h, the samples were incubated with 10 μM 2′,7′-dichlorofluorescin diacetate (DCFDA) (ab113851; Abcam, Cambridge, USA) in a light-protected humidified chamber at 37 °C for 30 min. Next, the sections were rinsed with PBS. The fluorescence intensity of DCFDA was measured by fluorescence microscopy (Olympus).

### Immunofluorescence staining

On the tenth day after injury, whole eyes were enucleated from the euthanized mice. The eyeballs in the eight groups were embedded in Tissue Tek OCT compound (Sakura Finetek) and sliced at a thickness of 7 μm using a Thermo microtome (Thermo). After rewarming the frozen cross sections at room temperature for 1 h, the sections were fixed in 4% (wt/vol) paraformaldehyde. The sections were blocked for 30 min with 5% BSA and incubated with primary antibodies at 4 °C overnight. The AIP1 and VEGFa levels were measured using mouse anti-AIP1 (1:100; Cat# sc-365921; Santa Cruz) and rabbit anti-VEGFa (1:100; Cat# A5708; ABclonal) primary antibodies, respectively. The sections were then incubated with secondary antibodies for 1 h at room temperature. One drop of HelixGen antifade fluorescence mounting medium containing 4′,6-diamidino-2-phenylindole was added to the sections. The sections were photographed under a fluorescence microscope (Olympus), and the area of neovascularization was analysed using ImageJ software.

### Immunoprecipitation

The 293 T cell line was purchased from Cellcook Co. Ltd. (#CC4003; Guangzhou, China) and cultured in a humidified atmosphere of 5% CO_2_ at 37 °C. The growth medium comprised Dulbecco’s modified Eagle’s medium (Gibco, Grand Island, USA) supplemented with 10% foetal bovine serum (Gibco). For immunoprecipitation in 293 T cells, the cells were transfected with plasmids encoding FLAG-NOX4 with or without Myc-AIP1 using Lipofectamine 2000 (Invitrogen, Boston, USA), and the lysates were harvested at 48 h posttransfection. The extracts were centrifuged, and the supernatants were used for immunoprecipitation with anti-Flag Magnetic Beads (Sigma–Aldrich, USA). After 6 h of incubation at 4 °C under rotation, the beads were washed 3 times with lysis buffer. Protein-magnetic bead complexes were eluted with 1 × SDS sample buffer and boiled for 10 min. The immunoprecipitates and samples of the input fractions were analysed by immunoblotting.

### Statistical analysis

After checking the normality using the Shapiro–Wilk test and SPSS 25.0 (IBM SPSS, Inc., Chicago, IL, United States), all the data were represented as means ± standard deviation (SD). One-way analysis of variance (ANOVA) with Dunnett’s multiple comparisons test was used to compare multiple groups, and Student’s t test was used to compare two groups. The rank sum test was used to compare hierarchical data between different groups. GraphPad Prism software (version 6.0.1; GraphPad Software Inc., San Diego, USA) was used to analyse the data. A *P* value less than 0.05 was considered to indicate statistical significance.

## Results

### AIP1 expression is markedly reduced after alkali burn injury

**Fig. 1:**
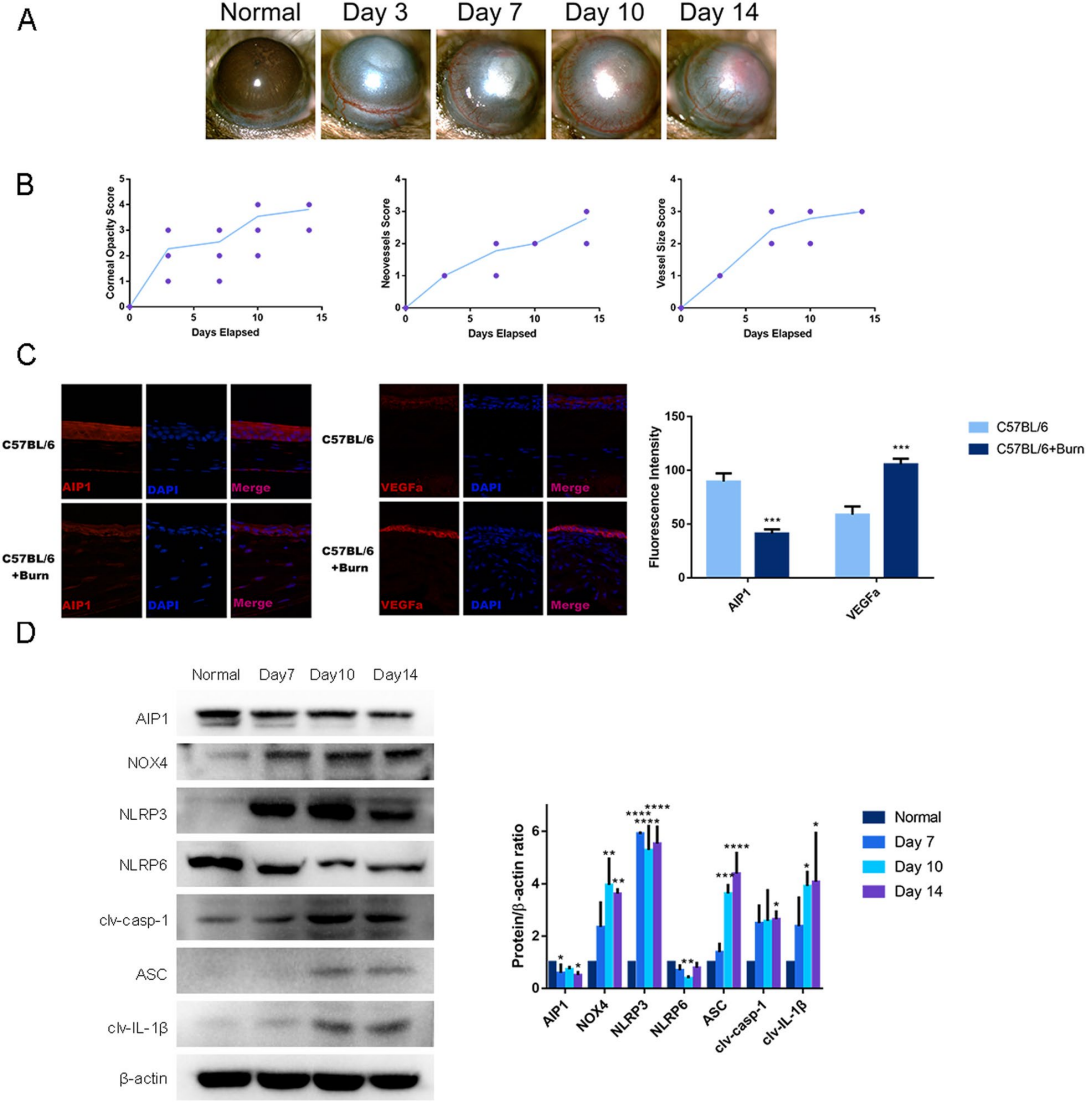
AIP1 expression is markedly reduced after alkali burn injury**. A** Representative slit-lamp images of the area of corneal neovascularization at different times after alkali burn injury (magnification: × 40). **B** Corneal opacity, neovessel size, and vessel size scores increased gradually from the third day of alkali burn injury and continued thereafter (N = nine). **C** Immunofluorescence staining revealed an obvious decrease in the expression of AIP1 and a concomitant increase in the expression of VEGFa after alkali burn injury (scale bar: 50 μm, magnification: × 400). **D** Western blot analysis demonstrating significantly upregulated expression of NOX4, NLRP3, ASC, clv-casp-1 and clv-IL-1β and downregulated expression of AIP1 and NLRP6 in the cornea after alkali burn injury (N = three). The error bars represent the means ± SD, and comparisons were made using one-way ANOVA. clv-casp1, cleaved caspase-1; clv-IL-1β, cleaved-IL-1β. *P < 0.05, **P < 0.01, ***P < 0.001, and ****P < 0.0001

First, we established an alkali burn murine model. Model mice demonstrated typical corneal neovascularization during alkali burn injury. The area of corneal neovascularization increased significantly from the third day of alkali burn injury and continued thereafter (Fig. 1A). Additionally, the corneal opacity, neovessel size, and vessel size scores increased gradually from the third day of alkali burn injury and thereafter (Fig. 1B). Immunofluorescence staining revealed an obvious decrease in AIP1 expression and a concomitant increase in VEGFa expression after alkali burn injury (Fig. 1C).

IL-1β participates in the progression of corneal neovascularization [13, 30, 31]. Western blot analysis demonstrated significantly upregulated expression of NOX4, NLRP3, ASC, cleaved caspase-1 (clv-casp-1) and clv-IL-1β and downregulated expression of AIP1 and NLRP6 in the cornea after alkali burn injury (Fig. 1D). These results suggest that the upregulation of clv-IL-1β and VEGFa due to inflammasome activation promotes the progression of corneal neovascularization after alkali burn injury.

### AIP1 depletion increases neovascularization, ROS production, and NOX4 expression and exacerbates the imbalance in NLRP3 activation and NLRP6 suppression

**Fig. 2:**
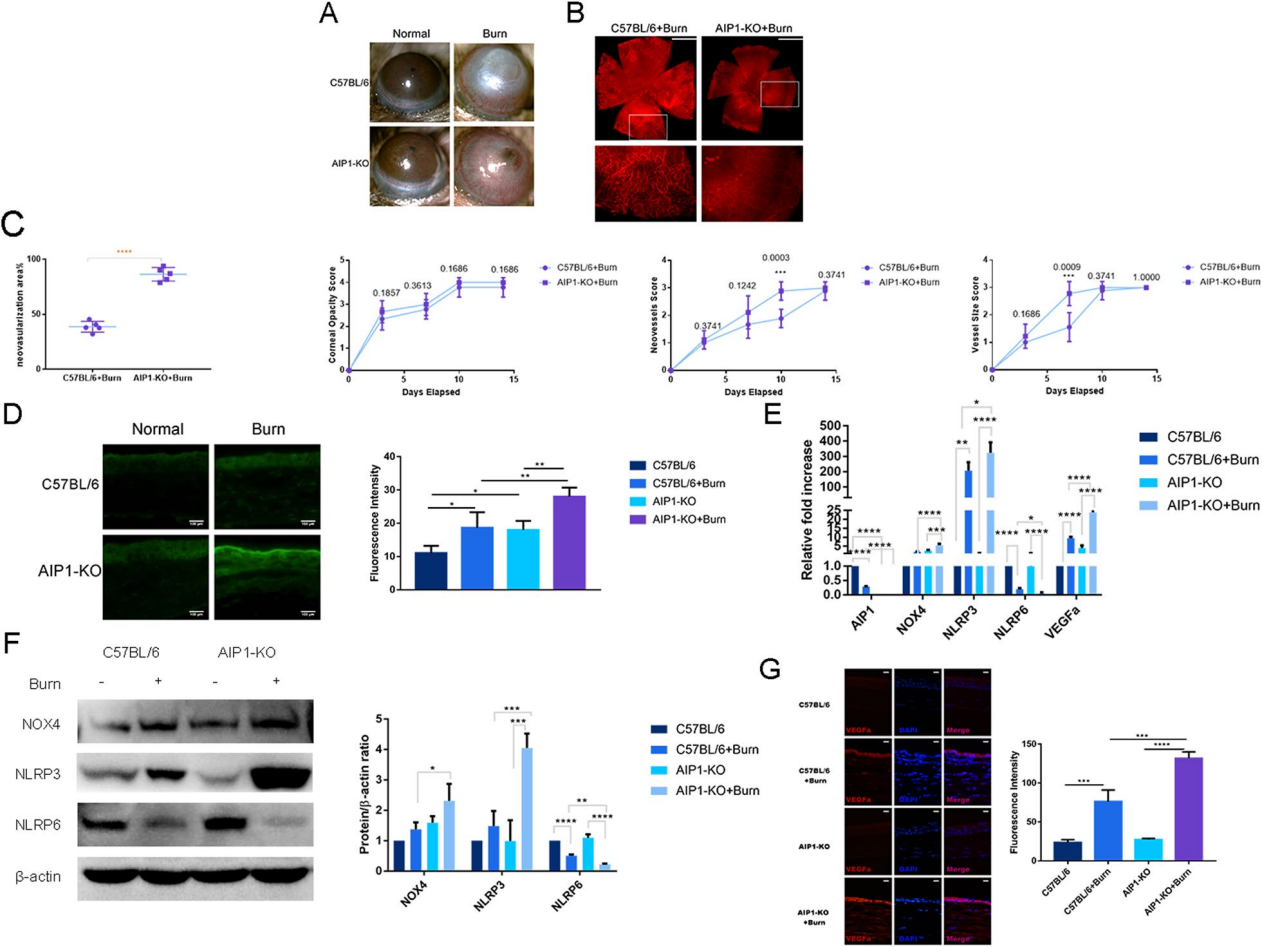
AIP1 depletion increases neovascularization, ROS production, and NOX4 expression and exacerbates the imbalance in NLRP3/NLRP6**. A** Representative slit-lamp images showing that AIP1 KO notably increased neovascularization compared with that in C57BL/6 mice (magnification: × 40). **B** Corneal whole-mount staining showing that AIP1 KO mice exhibited notably increased neovascularization compared with C57BL/6 mice (scale bar: 1 mm). **C** The corneal opacity, neovessel size, and vessel size scores increased significantly in AIP1-KO mice compared with those in C57BL/6 mice after alkali burn injury (N = nine). **D** The DCFDA ROS assay revealed that AIP1 KO notably increased ROS production after alkali burn injury compared with that in the control group (scale bar: 100 μm). **E** RT–qPCR analysis of AIP1 gene knockout efficiency. Deletion of AIP1 significantly increased the reduction in NLRP6 and elevation in NOX4, NLRP3 and VEGFa induced by alkali burn injury (N = three). **F** Western blot analysis showed that AIP1 deletion significantly increased the reduction in NLRP6 and elevation in NOX4 and NLRP3 induced by alkali burn injury (N = three). **G** Immunofluorescence staining showing that deletion of AIP1 significantly increased the elevation in VEGFa induced by alkali burn injury (scale bar: 50 μm; magnification: × 400). Error bars represent the mean ± SD, and comparisons were performed using one-way ANOVA. *P < 0.05, **P < 0.01, ***P < 0.001, and ****P < 0.0001

To examine the role of AIP1 in alkali burns, we compared corneal neovascularization in response to alkali burn injury in AIP1-KO mice and C57BL/6 mice. Slit-lamp images and corneal whole-mount staining revealed that AIP1 KO mice exhibited notably increased neovascularization compared with C57BL/6 mice (Fig. 2A, B). The corneas of AIP1-KO mice and C57BL/6 mice were both highly transparent and free of neovascularization (Fig. 2A). The scores for corneal opacity, neovessel size, and vessel size were higher in AIP1-KO mice than in C57BL/6 mice after alkali burn injury (Fig. 2C).

The DCFDA ROS assay revealed that AIP1 KO notably increased ROS production after alkali burn injury compared with that in the control group (Fig. 2D). AIP1 gene KO was confirmed by RT–qPCR analysis, which showed that AIP1 was significantly downregulated in AIP1-KO mice compared with that in C57BL/6 mice (Fig. 2E). AIP1 deletion significantly increased the reduction in NLRP6 induced by alkali burn injury at both the mRNA and protein levels (Fig. 2E, F). AIP1 deletion significantly increased the elevation in NOX4 and NLRP3 induced by alkali burn injury at both the mRNA and protein levels (Fig. 2E, F). Furthermore, AIP1 deletion significantly increased the elevation in VEGFa induced by alkali burn injury at both the mRNA and protein levels (Fig. 2E, G).

### AIP1 overexpression decreases neovascularization, ROS production, and NOX4 expression and alleviates the imbalance in NLRP3 activation and NLRP6 suppression

**Fig. 3:**
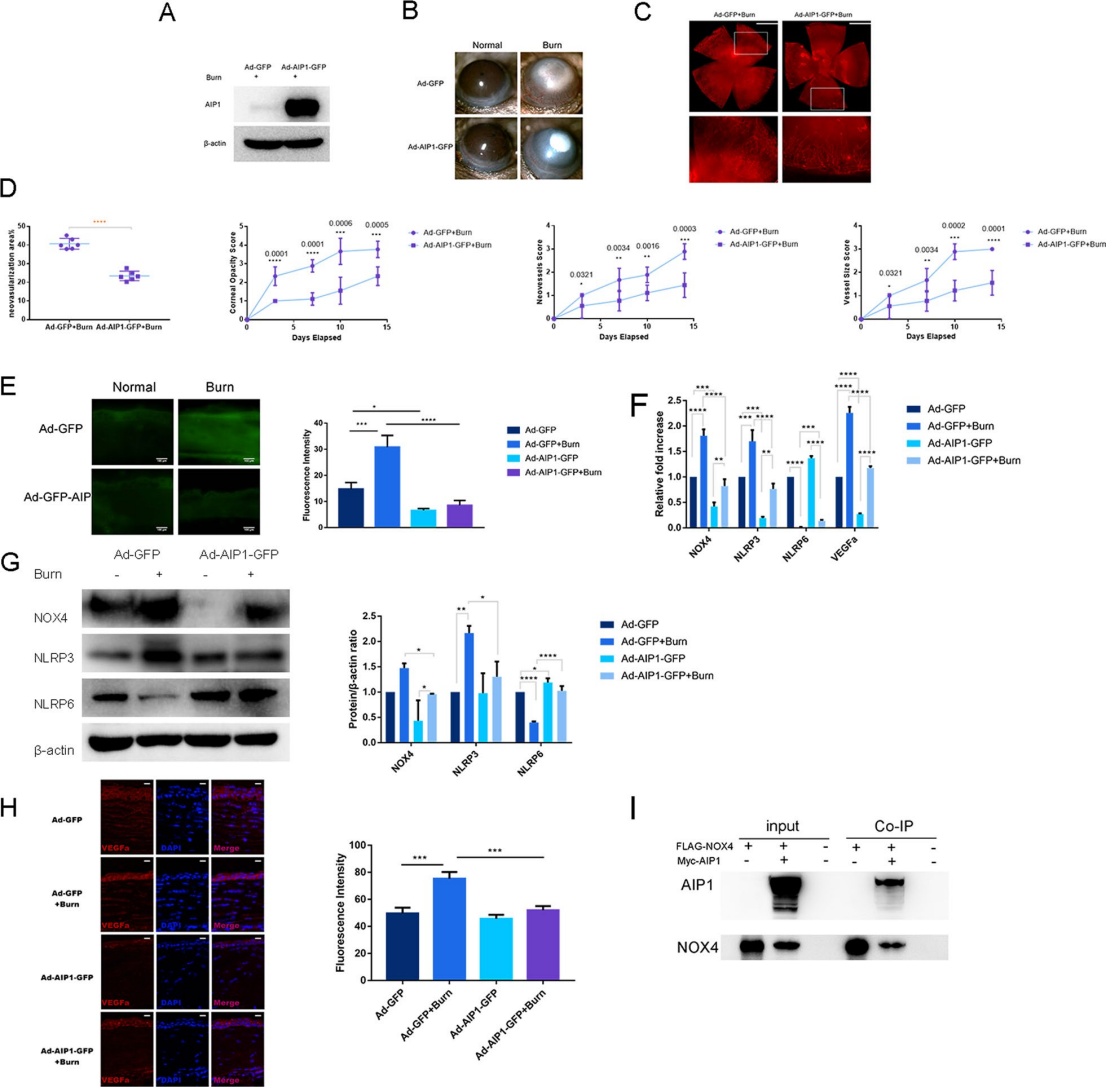
AIP1 overexpression decreases neovascularization, ROS production, and NOX4 expression and alleviates the imbalance in NLRP3/NLRP6. **A** Western blot analysis showing that AIP1 was significantly upregulated in AIP1-overexpressing mice compared with that in control mice after alkali burn injury (N = three). **B** Representative slit-lamp images showing that AIP1 overexpression notably decreased neovascularization compared with that in the control group (magnification: × 40). **C** Corneal whole-mount staining showing that AIP1 overexpression notably decreased neovascularization compared with that in the control group (scale bar: 1 mm). **D** The corneal opacity, neovessel size, and vessel size scores decreased significantly in AIP1-overexpressing mice compared with those in control mice after alkali burn injury (N = nine). **E** The DCFDA ROS assay revealed that AIP1 overexpression notably decreased the ROS production observed after alkali burn injury compared with that in the control group (scale bar: 100 μm). **F** RT–qPCR showed that AIP1 overexpression significantly abrogated the reduction in NLRP6 and decreased the elevation in NOX4, NLRP3 and VEGFa induced by alkali burn injury (N = three). **G** Western blot analysis showed that AIP1 overexpression significantly abrogated the reduction in NLRP6 and decreased the elevation in NOX4 and NLRP3 induced by alkali burn injury (N = three). **H** Immunofluorescence staining showed that AIP1 overexpression significantly decreased the elevation in VEGFa induced by alkali burn injury (scale bar: 50 μm; magnification: × 400). **I** Immunoprecipitation analysis showed that AIP1 could bind directly to NOX4 in 293 T cells (N = three). Error bars represent the mean ± SD, and comparisons were performed using one-way ANOVA. *P < 0.05, **P < 0.01, ***P < 0.001, and ****P < 0.0001

To verify the role of AIP1 in alkali burns, we compared corneal neovascularization in response to alkali burns in mice injected with Ad-AIP1-GFP in the anterior chamber and mice administered Ad-GFP. AIP1 gene overexpression was confirmed by western blotting, which showed that AIP1 was significantly upregulated in AIP1-overexpressing mice compared with that in control mice (Fig. 3A). Slit-lamp images and corneal whole-mount staining revealed that AIP1 overexpression notably decreased neovascularization compared with that in the control group (Fig. 3B, C). The corneas of AIP1-overexpressing mice and control mice were both highly transparent and free of neovascularization (Fig. 3B). The corneal opacity, neovessel size, and vessel size scores were lower in AIP1-overexpressing mice than in control mice after alkali burn injury (Fig. 3D).

The DCFDA ROS assay revealed that AIP1 overexpression notably decreased the ROS production observed after alkali burn injury compared with that in the control group (Fig. 3E). AIP1 overexpression significantly abrogated the reduction in NLRP6 induced by alkali burn injury at both the mRNA and protein levels (Fig. 3F, G). AIP1 overexpression significantly decreased the elevation in NOX4 and NLRP3 induced by alkali burn injury at both the mRNA and protein levels (Fig. 3F, G). Furthermore, AIP1 overexpression significantly decreased the elevation in VEGFa induced by alkali burn injury at both the mRNA and protein levels (Fig. 3F, H). Immunoprecipitation analysis further showed that AIP1 could bind directly to NOX4 in 293 T cells (Fig. 3I), suggesting that AIP1 might form a complex with NOX4 and decrease the activities of NOX4.

### The NOX4 inhibitor GLX351322 reverses the imbalance in NLRP3 activation and NLRP6 suppression

**Fig. 4:**
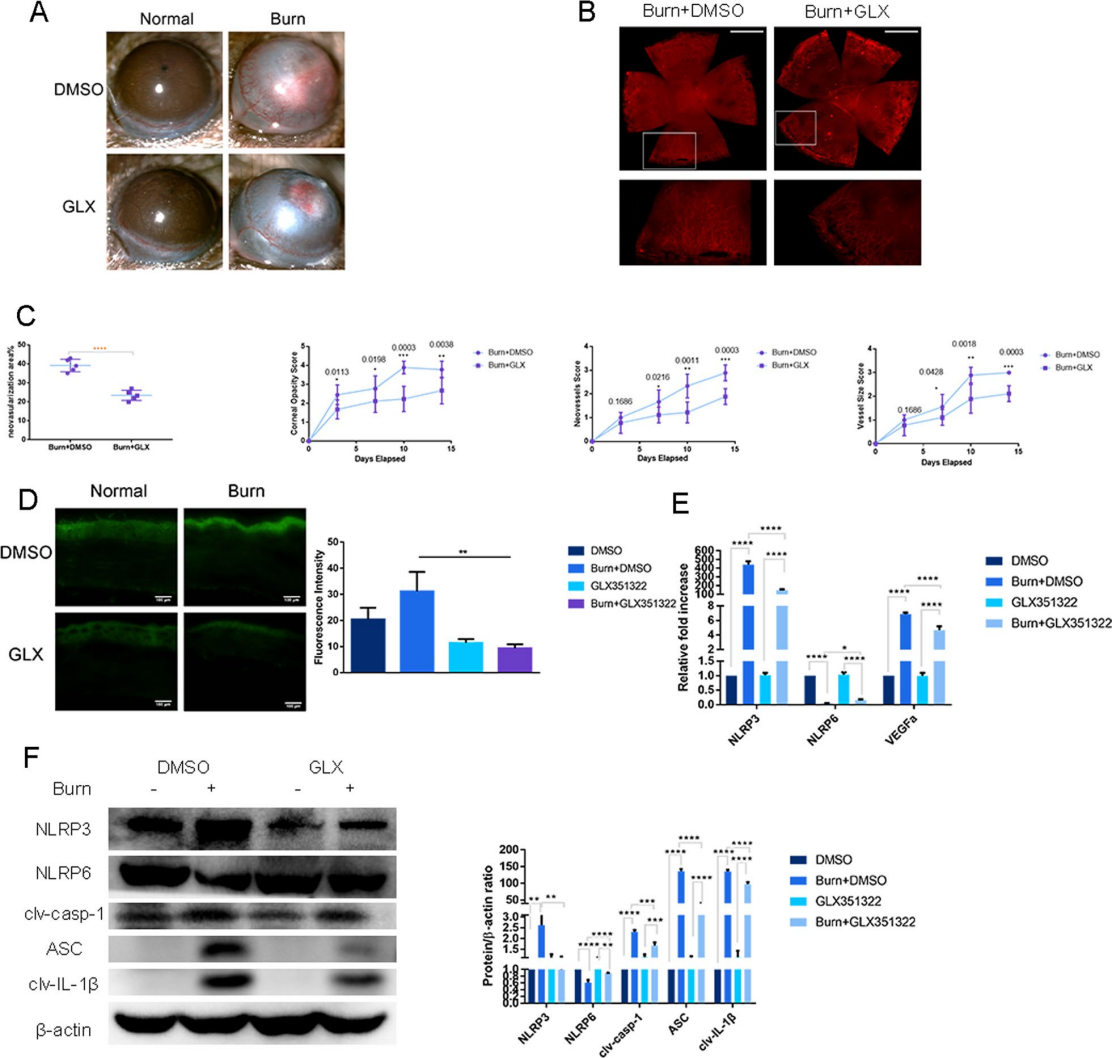
The NOX4 inhibitor GLX351322 reverses the imbalance in NLRP3 activation and NLRP6 suppression. **A** Representative slit-lamp images revealing that GLX351322 eye drops notably decreased neovascularization compared with that in the control group (magnification: × 40). **B** Corneal whole-mount staining revealed that GLX351322 eye drops notably decreased neovascularization compared with that in the control group (scale bar: 1 mm). **C** The corneal opacity, neovessel size, and vessel size scores decreased significantly in the GLX351322 eye drop groups compared with those in the control groups after alkali burn injury (N = nine). **D** The DCFDA ROS assay revealed that GLX351322 eye drops significantly reduced the increase in ROS induced by alkali burn injury compared with that in the control group (scale bar: 100 μm). **E** RT–qPCR showed that GLX351322 eye drops significantly abrogated the reduction in NLRP6 and decreased the elevation in NLRP3 and VEGFa induced by alkali burn injury (N = three). **F** Western blot analysis showed that GLX351322 eye drops significantly abrogated the reduction in NLRP6 and decreased the elevation in NLRP3, ASC, clv-casp1 and clv-IL-1β induced by alkali burn injury (N = three). The error bars represent the mean ± SD, and comparisons were performed using one-way ANOVA. clv-casp1, cleaved caspase-1; clv-IL-1β, cleaved-IL-1β. *P < 0.05, **P < 0.01, ***P < 0.001, and ****P < 0.0001

NOX4 expression is increased in the mouse cornea after alkali burns [22]. However, the specific molecular mechanisms by which NOX4 regulates corneal neovascularization require further exploration. Slit-lamp images and corneal whole-mount staining revealed that GLX351322 eye drops notably decreased neovascularization compared with that in the control group (Fig. 4A, B). The corneas in the GLX351322 eye drop and control groups were highly transparent and free of neovascularization (Fig. 4A). The corneal opacity, neovessel size, and vessel size scores were lower in the GLX351322 eye drop groups than in the control groups after alkali burn injury (Fig. 4C).

In the control groups, alkali burn injury induced oxidative stress, as demonstrated by increased ROS production. GLX351322 eye drops significantly reduced the increase in ROS (Fig. 4D). GLX351322 eye drops significantly abrogated the reduction in NLRP6 induced by alkali burn injury at both the mRNA and protein levels (Fig. 4E, F). GLX351322 eye drops significantly decreased the elevation in NLRP3 induced by alkali burn injury at both the mRNA and protein levels (Fig. 4E, F). GLX351322 eye drops significantly decreased the elevation in VEGFa induced by alkali burn injury at the mRNA level (Fig. 4E). Additionally, GLX351322 eye drops significantly decreased the elevation in clv-casp1, ASC and clv-IL-1β expression induced by alkali burn injury at the protein level (Fig. 4F).

### The protective effect of the NOX4 inhibitor and AIP1 on corneal neovascularization after alkali burn injury is associated with reduced ROS production and alleviated imbalance in NLRP3 activation and NLRP6 suppression

**Fig. 5:**
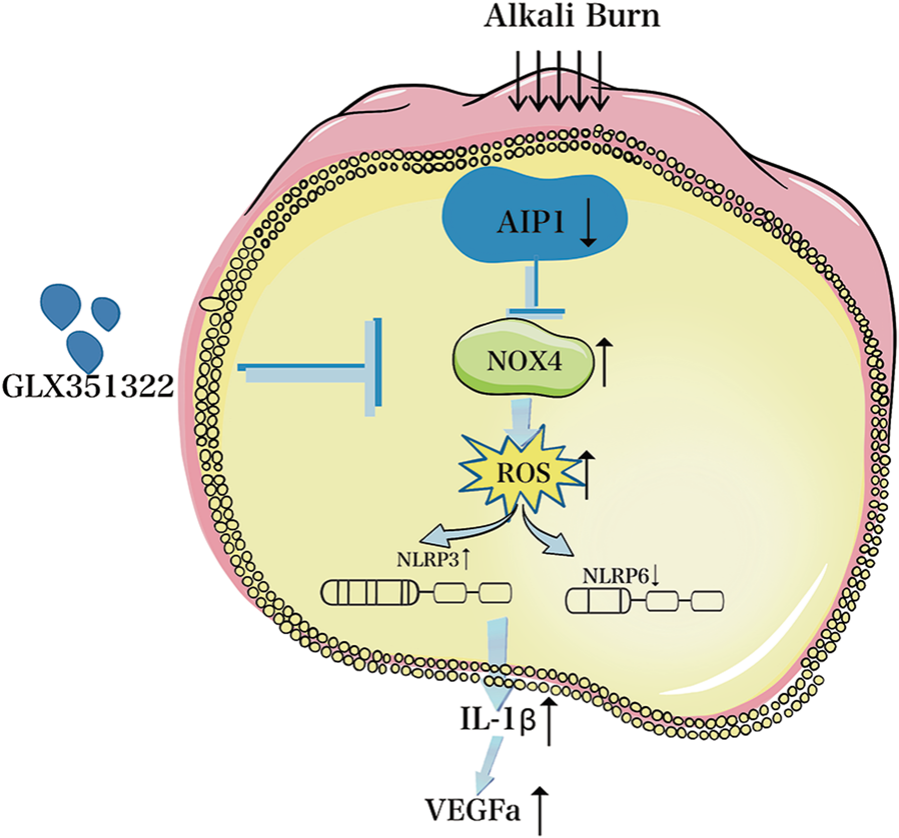
Schematic diagram of the proposed mechanism. The protective effect of the NOX4 inhibitor and AIP1 on corneal neovascularization after alkali burn injury is associated with a reduction in ROS production and alleviation of the imbalance in NLRP3 activation and NLRP6 suppression

The protective effect of GLX351322 on corneal neovascularization might be related to the reduction in ROS production, reversal of the imbalance in NLRP3 activation and NLRP6 suppression, and reduction in IL-1β and VEGFa production. AIP1 can exert the same effect as the NOX4 inhibitor GLX351322. Our results suggest that AIP1 and GLX351322 play important roles in protecting the cornea from inflammation and neovascularization caused by alkali burn injury and may be promising drugs for the treatment of corneal neovascularization caused by alkali burns (Fig. 5).

## Discussion

Corneal alkali burn injury is a common type of ocular injury that is difficult to treat in the clinic. Neovascularization after corneal alkali burn injury is a serious complication; currently, the best treatment for corneal neovascularization is transplantation. However, because of neovascularization and lymphangiogenesis, the incidence of immune rejection in corneal transplantation is remarkably high [1, 32]. Elevated levels of VEGF and IL-1β can exacerbate corneal neovascularization [13, 33], and IL-1β can induce the release of VEGFa [13]. The large amount of mature IL-1β produced by the innate immune response due to alkali burn injury may promote the release of VEGF and further exacerbate corneal neovascularization.

When tissues are stimulated by injury or hypoxia, damage-associated molecular patterns are released into the tissue space. Toll-like receptors or pattern recognition receptors, such as NLRs, then recognize and stimulate the innate immune system of the ocular surface [34–38]. NLRP3 and NLRP6 have been studied in various disease models. NLRP3 is present in human conjunctival and corneal epithelial cells [39], suggesting that NLRP3 is critical for immune regulation on the ocular surface. Our study showed that corneal epithelial cells during the pathogenesis of alkali burns activate NLRP3 and lead to a large release of IL-1β in the late stage of corneal neovascularization. Our study also showed that the normal cornea expresses high levels of NLRP6, and the expression of NLRP6 decreases for 2 weeks after alkali burn injury. This finding suggests that NLRP6 plays a protective role in corneas and that the decrease in NLRP6 promotes early inflammation and late neovascularization caused by alkali burns. We discovered an imbalance in NLRP3 and NLRP6 expression, with upregulated NLRP3 and downregulated NLRP6, caused by alkali burns. This result is consistent with previous findings that the oxidation of mitochondrial DNA disrupted the balance in the expression of NLRP3/NLRP6 in a dry eye murine model [40]. NLRP3 interacts with the ASC protein, which recruits pro-caspase-1 through its CARD domain to form inflammasomes [41]. NLRP3 activation leads to the self-cleavage of procaspase-1, which further cleaves pro-IL-1β and pro-IL-18 into biologically active IL-1β and IL-18, respectively. Alkali burn causes an imbalance in NLRP3 and NLRP6 expression in corneal epithelial cells, resulting in the production of substantial amounts of ASC, caspase-1, IL-1β, and VEGFa. Other studies have also shown that NLRP3 activation and the increase in IL-1β function as powerful proangiogenic factors in retinal or choroidal neovascularization [42, 43].

Under pathological conditions, ROS production and clearance are imbalanced. Studies have shown that the ROS-NLRP3-IL-1β signalling pathway plays a critical role in regulating inflammation [44–47]. TNF-α or alkali burns have also been reported to increase ROS production, triggering retinal or corneal neovascularization [48, 49]. The use of antiangiogenic treatments targeting ROS may address angiogenesis-related diseases [22, 50]. GLX351322 used in this study is a specific inhibitor of NOX4 compared with NOX2 [51]. The DCFDA kit used in this study mainly detects NOX4-derived hydrogen peroxide in corneas [52]. Increased NOX4 expression increases ROS production in response to stimulation by alkali burns. Our study showed that the specific NOX4 inhibitor GLX351322 could significantly reduce alkali burn-induced ROS production, reverse NLRP3/NLRP6 imbalance, and reduce the expression of IL-1β and VEGFa in corneal epithelial cells. These findings suggest that GLX351322 eye drops may abrogate alkali burn-induced oxidative stress and reverse subsequent NLRP3/NLRP6 imbalance. Additionally, we show that NOX4 regulates corneal neovascularization by regulating the innate immune response of the corneal epithelium induced by alkali burns or by directly upregulating VEGF expression through an ROS-dependent signalling pathway.

AIP1 inhibits tumour progression and metastasis by inhibiting VEGFR2-dependent signalling pathways [53]. Endothelial cell AIP1 regulates vascular remodelling by inhibiting NOX2 [26]. In the present study, we demonstrated that AIP1 KO exacerbated the pathological process, which was characterized by increased inflammation and neovascularization in a murine corneal alkali burn model. This effect was associated with enhanced NOX4 expression and ROS production in corneal tissue. Furthermore, AIP1 is a key negative regulator of NOX4. We showed enhanced NOX4 expression and increased ROS production after corneal alkali burns in AIP1-KO mice. Corneal inflammation and neovascularization after alkali burn injury were significantly inhibited by AIP1 overexpression. Immunoprecipitation analysis showed that AIP1 can bind directly to NOX4, suggesting that AIP1 may form a complex with NOX4 and decrease NOX4 activity. These data suggest that NOX4 is a major target of AIP1 in mouse corneas. Our experiments further investigated whether AIP1 affected corneal neovascularization by regulating the production of VEGF. Enhanced angiogenesis in AIP1-KO mice was associated with increases in the NOX4-NLRP3/NLRP6-IL-1β and VEGFa signalling pathways.

## Conclusions

Our study revealed that in addition to acting directly on VEGFR2, AIP1 could directly inhibit corneal neovascularization by reducing VEGFa production. Our findings suggest that NOX4 may mediate the interaction of AIP1 with inflammasomes and highlight the critical role of AIP1 as an anti-inflammatory protein. Furthermore, inducing the expression of AIP1 with gene therapy is a potential therapeutic approach to treat corneal neovascularization diseases.

## Data Availability

All other data supporting the findings of this study are available from the corresponding authors upon reasonable request.

## Abbreviations

AIP1: ASK1-interacting protein-1
NLRs: Nucleotide-binding oligomerization domain (NOD)-like receptors
NLRP3: NLR Family Pyrin Domain Containing 3
NLRP6: NLR Family Pyrin Domain Containing 6
NOX4: NADPH oxidase 4
ROS: Reactive oxygen species
Clv-casp-1: Cleaved caspase-1
clv-IL-1β: Cleaved interleukin-1β
VEGFa: Vascular endothelial growth factor A
GAPDH: Glyceraldehyde 3-phosphate dehydrogenase
RT–qPCR: Quantitative Real-Time Polymerase Chain Reaction
